# Evaluating Sodium Restriction in Heart Failure

**DOI:** 10.5935/abc.20190017

**Published:** 2019-02

**Authors:** Pedro Pimenta de Mello Spineti

**Affiliations:** 1 Hospital Universitário Pedro Ernesto, Rio de Janeiro, RJ - Brazil; 2 Hospital Unimed-Rio, Rio de Janeiro, RJ - Brazil

**Keywords:** Heart Failure/complications, Diet-Sodium- Restricted/methods, Diet Therapy, Treatment Adherence and Compliance, Patient Compliance, Survey and Questionnaires

Although salt and water retention plays a crucial role in heart failure (HF) pathophysiology, controversy still exists about dietary salt restriction in the treatment of HF patients.^1^ Small clinical studies have suggested that excessive sodium restriction (< 5 g of salt per day), as compared with normal-sodium diet (approximately 7 g of salt per day), may be associated with deleterious effects in patients with chronic HF, including increased neurohormonal activation, and higher hospitalization and mortality rates.^2,3^

A recent meta-analysis^4^ of nine studies involving 479 HF patients undergoing dietary sodium restriction was inconclusive for the recommendation of this strategy in hospitalized patients. None of the studies analyzed in the meta-analysis included hard endpoints such as all-cause death or cardiovascular mortality. However, a modest tendency for improvement of functional class was observed in outpatients undergoing sodium restriction intake. The author reinforces the need for randomized, prospective studies including large sample sizes, evaluating the effect of different regimens of sodium intake on relevant outcomes to build evidence base for detailed recommendations.

Restriction of sodium intake - < 3 g/day or < 7 g/sodium chloride (table salt) - is one of the non-pharmacological measures recommended by the Brazilian Guidelines on Heart Failure^1^ and by the American Heart Association^5^ (AHA) guidelines. The AHA also recommends evaluating patient understanding and the level of water and sodium intake restriction, as well as educating patients to reduce sodium intake.

However, compliance with this recommendation remains challenging. In 2009 Bentley et al.^6^ proposed the adoption of a new instrument, the Dietary Sodium Restriction Questionnaire (DSRQ), aimed at measuring attitude, beliefs and barriers of symptomatic HF patients (NYHA II/III) in following a low-sodium diet. Based on the Theory of Planned Behavior, the questionnaire assesses adherence through three subscales: attitude, subjective norm, and perceived behavioral control.

D’Almeida et al.^7^ adapted the DSRQ to the Brazilian population in 2012,^7^ and showed its validity and reliability in 2013.^8^ The Brazilian version of the DSRQ is composed of 27 items, 11 descriptive questions and 16 questions divided into three subscales: attitude and subjective norm, perceived behavioral control, and dependent behavior.

In this issue of Arquivos Brasileiros de Cardiologia, the same authors proposed the determination of a cut-off point to evaluate adherence to a low-sodium diet in Brazilian patients with HF. This was a case-control study that compared the scores of each subscale between 206 outpatients with compensated HF and 255 patients with uncompensated HF. Mean application time of the instrument was 40 minutes. The best area under the ROC curve was observed for the attitude and subjective norm scale (0.725). The cut-off for this subscale was 40 out of 45 points, with a 53.8% sensitivity and 82.5% specificity.

Previous studies had already shown an association between subjective norm subscale and an increased sodium urinary excretion^9^ and that the attitude subscale is the only associated with long-term adherence (six months),^10^ which corroborate the validity of their results. The proposed cut-off points to measure adherence to a low-sodium diet can be useful for future longitudinal studies aiming at elucidating the role of sodium restriction in the treatment of patients with HF.
